# Deep Learning Design for Loss Optimization in Metamaterials

**DOI:** 10.3390/nano15030178

**Published:** 2025-01-23

**Authors:** Xianfeng Wu, Jing Zhao, Kunlun Xie, Xiaopeng Zhao

**Affiliations:** 1 Smart Materials Laboratory, Department of Applied Physics, Northwestern Polytechnical University, Xi’an 710129, China; shingfer@mail.nwpu.edu.cn (X.W.); klxie@mail.nwpu.edu.cn (K.X.); 2 Medtronic PLC, Boulder, CO 80301, USA

**Keywords:** metamaterial, disordered dispersion, loss optimization, deep learning

## Abstract

Inherent material loss is a pivotal challenge that impedes the development of metamaterial properties, particularly in the context of 3D metamaterials operating at visible wavelengths. Traditional approaches, such as the design of periodic model structures and the selection of noble metals, have encountered a plateau. Coupled with the complexities of constructing 3D structures and achieving precise alignment, these factors have made the creation of low-loss metamaterials in the visible spectrum a formidable task. In this work, we harness the concept of deep learning, combined with the principle of weak interactions in metamaterials, to re-examine and optimize previously validated disordered discrete metamaterials. The paper presents an innovative strategy for loss optimization in metamaterials with disordered structural unit distributions, proving their robustness and ability to perform intended functions within a critical distribution ratio. This refined design strategy offers a theoretical framework for the development of single-frequency and broadband metamaterials within disordered discrete systems. It paves the way for the loss optimization of optical metamaterials and the facile fabrication of high-performance photonic devices.

## 1. Introduction

Optical metamaterials offer unparalleled control over light, enabling manipulation at sub-wavelength scales; however, metamaterials based on noble metal structures suffer from considerable ohmic losses and bandwidth limitations [1,2,3,4]. Although some dielectric materials with transparent windows in the visible band (such as gallium nitride, silicon nitride, and titanium dioxide) have been used to develop low-loss photonic devices, such as high-efficiency meta-lenses [5,6], manufacturing low-loss 3D negative index metamaterials in the visible spectrum has been a difficult problem due to the complexity of 3D meta-atom structures. The performance of metamaterials is predominantly hindered by unit structure losses, which can lead to reduced functionality and efficiency [7,8], such as diminished meta-lens resolution [3,4]. Various models and techniques have been proposed to mitigate these losses and enhance performance [9,10,11,12]. Metasurfaces [13,14], a “reduced-dimensional” variant of metamaterials with ultrathin profiles, have been effective in minimizing loss. Additionally, all-dielectric metasurfaces, designed with a high refractive index and low-loss dielectrics, have emerged as a promising solution for high-efficiency light modulation [15,16,17,18,19,20], adeptly circumventing high ohmic damping [21,22,23,24]. Recent research on complex frequencies [3,4] has demonstrated significant effects in reducing losses by cleverly constructing Fourier transforms that convert between integration and summation, synthesizing equivalent complex frequencies through discrete frequency points. However, lithography, the prevalent fabrication method for metamaterials, remains time-consuming, costly, and limited in its ability to produce complex 3D structures, hindering large-scale production [25]. While metasurfaces simplify fabrication and foster new applications, they still fall short in realizing functionalities that require intricate 3D structures due to the restricted extent of light–matter interactions [26].

The design of 3D supramolecular structures remains a formidable task, particularly when it comes to controlling nanogaps as their numbers increase. The traditional manual trial-and-error approach to metamaterial design may no longer suffice, making it particularly challenging to achieve negative refractive indices in metamaterials assembled from colloids [25]. To address the issue of metamaterial loss in the visible spectrum, we have proposed and validated a series of low-loss metamaterials [27,28,29,30]. The metamaterial poly(amidoamine) (PAMAM)-Ag [27] leverages PAMAM as both a template and stabilizer, allowing for the photochemical reduction and growth of silver nanoparticles within its molecular cavities. Experiments have demonstrated that the fifth-generation (5G) PAMAM-Ag metamaterial is capable of generating negative Goos–Hänchen (GH) shifts and an anomalous spin Hall effect of light (SHEL) across a broad visible spectrum, effective over a wide range of incidence angles. Furthermore, the 5G PAMAM-Ag planar lens has been shown to achieve efficient focusing within the 750–1050 nm wavelength range. Additionally, we have crafted a novel ball-thorn-shaped metamaterial model, referred to as a meta-cluster [28], which is composed of a spherical core and hundreds of protruding rods, resembling the structure of a ciliated cell. Both the spherical nuclei and rods are composed of TiO_2_, which is coated with a 1 nm thick layer of silver. Prepared using a bottom-up synthesis strategy, this meta-cluster has been experimentally shown to produce negative refractive indices and to exhibit an inverse Doppler effect at red and green light frequencies. It has been demonstrated that these meta-clusters possess a high figure of merit (FOM), indicating their low-loss characteristics. This meta-cluster design is independent of the previously widely used meta-atom design, and the model greatly reduces the thickness of the silver coating required to achieve a high FOM. This innovation provides a physical foundation for diminishing Joule heating, thereby facilitating ultra-low loss performance, and results in an FOM nearly an order of magnitude superior to that of current technologies. Building upon this meta-cluster model and the weak interaction theory, we have engineered a visible broadband metamaterial [29], which is composed of randomly assembled, narrow-band, omnidirectional, and ultra-low-loss meta-clusters. For the first time, we have observed a negative refractive index within the 490 nm–730 nm band and a broadband inverse Doppler effect that covers most of the visible spectrum on a 3D large-area sample. With this broadband metamaterial, we have also observed the negative Goos–Hänchen effect across the visible spectrum and discovered a broadband amber rainbow effect and optical black holes in axially varying inhomogeneous waveguides [30]. It is noteworthy that all the aforementioned metamaterials exhibit quasi-periodic structures, which complicates the achievement of complete equivalence in the simulation design. Although we have experimentally demonstrated these metamaterials and achieved remarkable optical properties, such works are insufficient to provide a universal paradigm for broader metamaterial design and application. By leveraging deep learning concepts for analysis, advanced computational optimization tailored to disordered discrete structures can offer more precise guidance for the fabrication of metamaterials. This guidance can help simplify the preparation process and optimize losses, thereby striking an exquisite balance between the design, fabrication complexity, and performance of metamaterials.

Recent studies have shown that micro- and nano-structures with engineered disorder between perfect order and complete randomness can give rise to a variety of new phenomena in optics [31,32,33]. Metasurfaces based on disorder engineering exhibit unprecedented degrees of freedom in wavefront shaping and additional unique features. The computation of disordered discrete metamaterials needs to be further clarified and will significantly contribute to advances in metamaterial design. As one of the most important branches of machine learning, deep learning has become a fundamental way to learn complex hierarchical features. Inspired by the development of AI technology, the intelligent design of materials and their performance prediction have received extensive attention from researchers [34,35]. The deep integration of deep learning and nanophotonics has been widely reported in the literature [36,37], and it offers significant advantages for the fast prediction and optimization of complex systems. Overall, in order to find the final optimized design of 3D metamaterials, deep learning design based on neural networks may provide a new boost to this rapidly growing field.

Drawing inspiration from the field of disordered engineering and discrete systems, we have conducted an analysis of existing disordered metamaterial systems and identified potential for further design optimization. Specifically, we have discovered that by fine-tuning the ratio of disordered and discretely distributed units within metamaterials, and by employing deep learning concepts to streamline the analytical and computational processes, significant advancements can be made. In this work, we introduce a novel method for metamaterial loss optimization, focusing on the design of discrete distribution units. By scrutinizing the original 2D “wire and split-ring” unit array and extending our analysis to a 3D ball-thorn-shaped metamaterial model, we have harnessed the power of deep learning models to achieve our goals. Our findings indicate that it is possible to substantially reduce losses in metamaterials without compromising their inherent properties by selectively discretizing or partially removing units. This innovative loss optimization design method diverges from conventional approaches to structural and material optimization. Instead, it takes a fresh perspective by focusing on the disordered, discrete distribution of units, offering a pathway for loss optimization. This breakthrough paves the way for the development of high-performance metamaterials and the design of sophisticated photonic devices, marking a significant step forward in the field.

## 2. The Wire and Split-Ring Model

**Figure 1:**
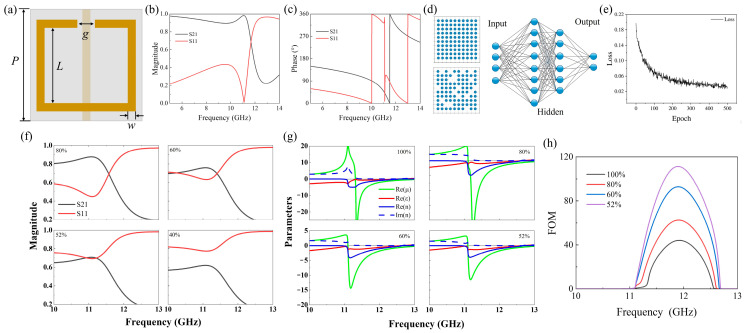
Optimization design of “wire and split-ring” metamaterials: (**a**) “wire and split-ring” model. (**b**) the coefficients and (**c**) phase of transmission and reflection spectra. (**d**) array design and schematic diagram of a fully connected feed-forward neural network. (**e**) learning losses for fully connected feed-forward neural networks. (**f**) the transmission and reflectance spectra of the “wire and split-ring” model with 80%, 60%, 52%, and 40% effective unit arrays. (**g**) effective electromagnetic parameters with 100%, 80%, 60%, and 52% effective unit arrays. (**h**) FOM corresponding to (**f**).

Theoretical models of metal wires can produce negative permittivity [38], and metal split-ring resonators can produce effective negative permeability [39]. Such “wire and split-ring” units were the first fundamental metamaterials to experimentally confirm negative refraction [40]. Here, a “wire and split-ring” model is taken as an example, as shown in Figure 1a. The structural parameters are *P* = 2.5 mm, *L* = 1.8 mm, *w* = 0.2 mm, and *g* = 0.3 mm. The wire is located at the center on the back side of the dielectric substrate, with a length of *P* and a width of *w*. The thickness of the substrate is 0.2 mm and the permittivity is 4.4. The structure is made of metallic silver with a thickness of 0.017 mm. Its transmission and reflection spectra (Figure 1b,c) are obtained by CST Microwave Studio simulation (Appendix A), and based on them, the effective electromagnetic parameters can be extracted.

Within a limited range, it is assumed that the complete metamaterial is a 100 × 100 periodic array (Figure 1d, upper left panel), and its electromagnetic parameters are calculated as shown in Figure 1b,c. We can consider the effective parameters of the metamaterial in case of defects in the unit array, e.g., only 80% of the array is present (Figure 1d, lower left panel). According to the relevant literature [41,42,43], the transmission spectra of multiple samples can be equated to the overlap of a single spectrum for each sample. Therefore, the formulas for the transmission and reflection coefficients of multiple units can be derived:

(1) $$\begin{array}{l} R=\frac{1}{n}\sum_{i}^{n-m}f_{i}R_{i}\\ T=\frac{1}{n}\left(\sum_{i}^{n-m}f_{i}T_{i}+m\right) \end{array}$$

where *n* is the total number of cell arrays, *m* is the number of defects, and *f_i_* is the modified distribution factor.

Deep learning models have been used to establish a mechanism corresponding to the structural parameters and behavioral features of metamaterials, which can quickly predict the spectral behavior from the input parameters, known as forward prediction. By combining simulation datasets with deep learning networks, metamaterials with specific parameters can be quickly retrieved on demand. In this work, the deep learning modeling platform used is TensorFlow (version 2.10.0, with a dependent CUDA version of 11.2). A fully connected feed-forward neural network (FcFNN) has been established, and the purpose of training is to build a deep learning network for learning the mapping from structural parameters to electromagnetic responses. The input layer of the FcFNN consists of a fully connected neural layer with four neurons, each corresponding to the structural parameters of unit occupancy ratio *ω*, structural line length *L*, line width *w*, and periodic parameter *P*, as shown in Figure 1d. To ensure that the input parameters are not distorted, neurons without nonlinear activation functions are used in this layer. To obtain the training dataset, the structural parameter matrix serves as the input data, while the transmission and reflection coefficients serve as the output data. These combined datasets are calculated through electromagnetic field simulation using CST, and are constituted by matching the structural parameters with the transmission and reflection coefficient curves one by one. After preliminary processing, we allocate 70% of the data set as the training set, 15% as the test set, and the remaining 15% as the validation set. In the FcFNN, it can also be seen that the network model includes four hidden layers, which constitute the main body for implementing the deep learning function. We use the Adam optimizer with a mean squared error loss function to train the network, as the output matrix contains transmission and reflection coefficients, which are not sparse matrices, and thus cannot use the cross-entropy loss function. The training batch size is 32, the number of epochs is 500, and the learning rate is 0.001. The training of this network model gradually converges after 200 epochs, with a final loss rate of about 4% (see Figure 1e). After training, the model is further evaluated using the validation set, and the accuracy of the FcFNN on the validation set is 91%. Based on the deep learning approach, we have established a fast retrieval mechanism to quickly retrieve the transmission and reflection spectra by specifying the unit occupancy ratio *ω*. The results for the “wire and split-ring” model array when *ω* is 80%, 60%, 52%, and 40% are given as shown in Figure 1f. The effective electromagnetic parameters extracted from spectral results are shown in Figure 1g; the model can be found to produce a negative refractive index near 11 GHz. As a constraint for retrieval, when the distribution of the transmittance/reflection spectra exceeds the critical state (*ω =* 40% in Figure 1g), the effective parameters cannot be retrieved due to too many missing effective units, and it is considered to be ineffective. The maximum value of FOM can be obtained at an array distribution ratio of 52% (Figure 1h).

The above results show that metamaterial arrays are robust to defects and disorder, which can produce anomalous behaviors, provided that the unit distribution ratio of the disordered system satisfies the critical range. For the “wire and split-ring” array, the metamaterial system can operate normally and significantly reduce losses when the array distribution ratio exceeds the critical value of 52%. Based on theoretical calculations, the unit ratio can be optimized to meet the need to reduce losses of metamaterials.

## 3. PAMAM-Ag Metamaterial

**Figure 2:**
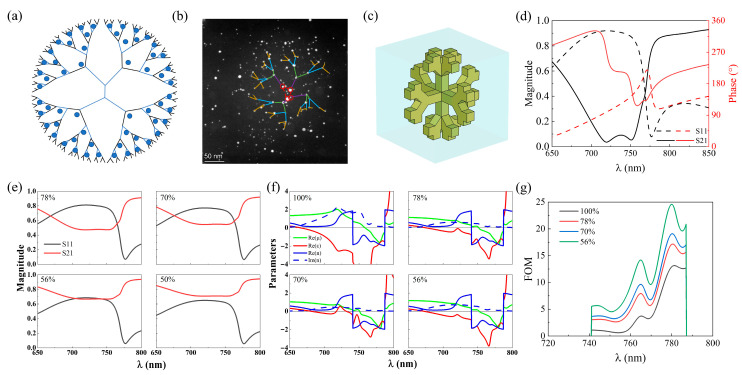
Optimization design of metamaterial PAMAM-Ag. (**a**) PAMAM-Ag Model [27]. (**b**) HAADF-STEM image. The red circles mark the Ag nanoparticles in the centre, and the colored lines represent different levels. (**c**) Three-dimensional silver dendrite model. (**d**) The coefficients and phase of transmission and reflection spectra obtained by simulation. (**e**) The transmission and reflectance spectra for the 3D silver dendrite model with 78%, 70%, 56%, and 50% effective unit ratio. (**f**) Effective electromagnetic parameters for the 3D silver dendrite model with 100%, 78%, 70%, and 56% effective unit ratio. (**g**) FOM corresponding to (**f**).

The metamaterial PAMAM-Ag [27] is a composite material consisting of silver nanoparticles and dendrimer macromolecules, and is obtained by coordinating silver ions with nitrogen atoms at the nodes of the dendrimer macromolecules and then reducing them to silver monomers. As shown in Figure 2a,b, Ag grows by attaching to the dendrimer cavity. During the reaction, the dendrimers act as “network containers” for the capture of silver ions and as reagents for electron transfer, and Ag^+^ is immobilized by the “N” atoms of the PAMAM. Reduced silver nanoparticles obtained in the fifth-generation PAMAM are approximately 9 nm in size and are distributed in a multilayered dendritic pattern in 3D. Due to the complexity of the model, we replace the PAMAM-Ag model with a 3D distributed silver dendrite model (Figure 2c). The 3D silver dendrite model can be regarded as a collection of multilevel effective units, and its transmission and reflection spectra are calculated as in Figure 2d.

Based on the calculations, a 3D silver dendrite model is considered to contain 54 effective units. Similarly, the transmission and reflection spectra of silver models with 78%, 70%, 56%, and 50% effective unit ratios were retrieved by establishing a feedback mechanism, as shown in Figure 2e. The effective electromagnetic parameters and FOM extracted from spectral results are shown in Figure 2f,g, and this model can operate in the red light wavelength, in agreement with the previous experimental results. The maximum value of FOM can be obtained at an array distribution ratio of 56%. When the effective unit ratio is lower than 56%, the transmission/reflection spectral distribution exceeds the critical state (as shown in Figure 2e) due to too many defects, and the effective parameters cannot be retrieved, rendering the metamaterial ineffective. It can be found that the loss can be significantly reduced by appropriately adjusting the defective portion of the branch units without impairing the negative refraction performance.

The morphological characterization shown in Figure 2b illustrates that the metamaterial PAMAM-Ag is actually distributed in a disordered manner. Both experimental results [27] and theoretical analysis show that metamaterial PAMAM-Ag can be distributed in a disordered manner and that silver nanoparticles do not necessarily fill all branches of the model. The analysis shows that there exists a critical state for the disordered distribution that allows for the realization of metamaterial properties, forming the best optimized state after a threshold is reached. Both the negative GH effect and the anomalous spin Hall effect of light are extremely small shifts at material interfaces, which are difficult to observe and have only been reported in a few articles on experimental measurements so far [44,45,46]. Benefiting from this loss-optimized metamaterial, we experimentally measured the previously unattainable weak measurement state, including the negative GH effect and the anomalous spin Hall effect [27].

## 4. Ball-Thorn-Shaped Metamaterials

**Figure 3:**
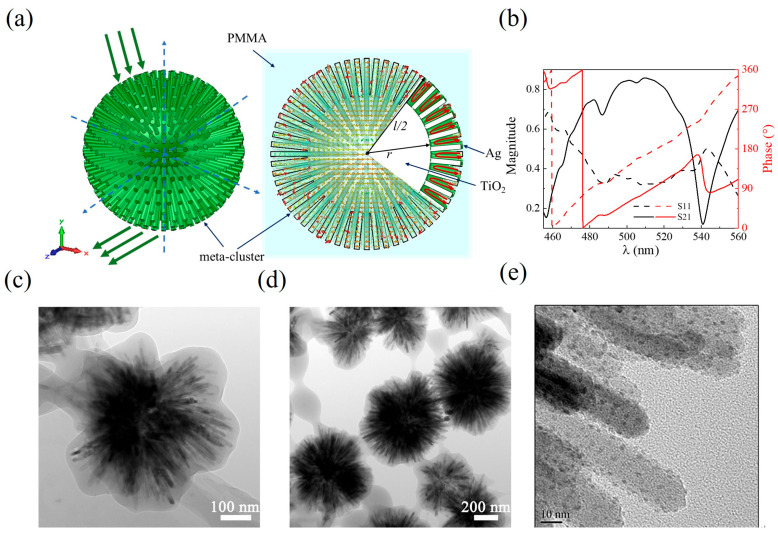
Green light meta-cluster model and sample morphology [28]. (**a**) Green light meta-cluster model. The green arrows indicate incident and outgoing light. (**b**) simulated transmission and reflection spectra. (**c**–**e**) TEM images of the meta-cluster samples.

In response to the high loss and anisotropy issues of visible metamaterials, we have designed a ball-thorn-shaped metamaterial model (meta-cluster) [28,29,30] inspired by the structure of ciliated cells, which consists of a spherical nucleus and hundreds of protruding rods (Figure 3a). The spherical nucleus and rods are composed of TiO_2_, which is covered with a 1 nm thick layer of Ag. In fact, each meta-cluster can be viewed as consisting of 1200 meta-atoms: the U-shaped split-ring and the equivalent wire are uniformly and symmetrically distributed in space. The geometrical dimensions of these meta-atoms are much smaller than the wavelength, and it is their independent resonance that forms the outfield resonance response of the meta-cluster (Appendix B). The proposed metamaterial particles are prepared by the solvothermal synthesis method, which can be easily realized by experiments. To address the problem of nano silver layer coating of meta-clusters, a certain amount of AgNO_3_ is first mixed into TiCl_4_ to form AgCl during the preparation of TiO_2_ cores. After the photoreduction, AgCl is further decomposed into elemental chlorine and metallic silver, with the latter precipitating on the surface of the TiO_2_ spherical nucleus to form a discretely distributed silver layer approximately 1 nm in thickness. The whole structure is then protected by PMMA wrapping, and the final nanostructure is named Ag/AgCl/TiO_2_@PMMA. Silver is discretely distributed on the surface of the meta-cluster and can produce plasma resonance when excited by electromagnetic waves, thus realizing the properties of metamaterials. The meta-cluster particles can be disorderedly self-assembled into wedge-shaped blocks and single-layer film samples. Using the block samples, the negative refractive index and inverse Doppler effect in the visible light band are directly measured in the experiment [28]. Furthermore, the spherically symmetric structure of the meta-clusters makes them perfect candidates for self-assembly, and the metamaterial achieves a 3D omnidirectional response with ultra-low loss. In the initial simulations, a continuous silver layer design was used. Figure 3a shows the meta-cluster model operating at the green light wavelength, and the simulated transmittance and reflectance spectra are shown in Figure 3b. Figure 3c–e show the SEM images of the green light sample and the red light sample.

**Figure 4:**
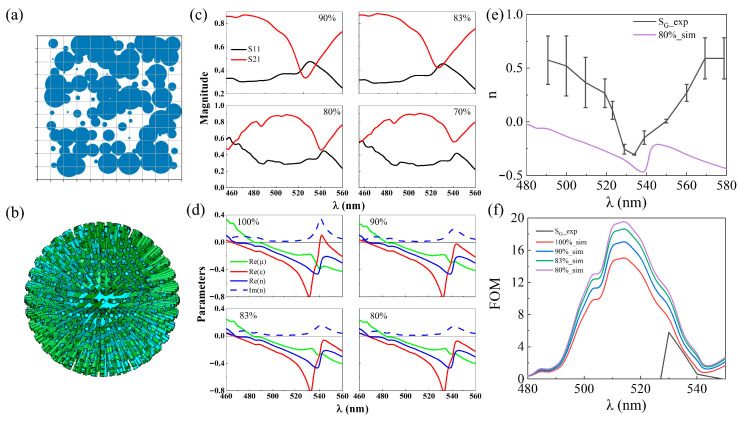
Optimization design of green light meta-cluster. (**a**) Schematic diagram of discrete distributed silver layer. (**b**) meta-cluster model. (**c**) the transmission and reflectance spectra of the green light meta-cluster model with 90%, 83%, 80%, and 70% silver layer distributions. (**d**) effective electromagnetic parameters for the green light meta-cluster model with 100%, 90%, 83%, and 80% silver layer distributions. Comparison of experimental and simulation results for green light meta-cluster sample with (**e**) refractive index and (**f**) FOM.

By analogy with the aforementioned analysis, the number of effective units affects the actual results. Since the silver in the actual prepared samples is discretely distributed, the effective number of units is less than 1200, and the discretely distributed silver layer with the meta-cluster model is shown in Figure 4a,b. In the calculations, the proportion of the discrete silver layer is taken into account as a parameter, which can also be translated into the number of rods for the calculations. Similarly to the derivation process above, we retrieved the transmission and reflection spectra for meta-cluster models with silver distributions of 90%, 83%, 80%, and 70%, as shown in Figure 4c. The model maintains negative refraction performance when the silver distribution is greater than 70% (Figure 4d,e) and achieves the maximum FOM when the distribution ratio is 80% (Figure 4f). When the ratio is lower than 80%, the transmission and reflection curves are separated and the correct effective electromagnetic parameters cannot be extracted.

It is worth noting that it is the distribution of the surface silver layer that is considered in the meta-cluster metamaterial and its absolute metal content is much smaller than that of the actual split-ring model and the PAMAM-Ag model. Figure 4e,f illustrate the experimentally measured refractive index and FOM values of the green light meta-cluster sample [28], as well as a comparison with the predicted results. It can be observed that there are discrepancies between the experimental results and the predicted results, and these discrepancies stem from the irregularity of the ball-thorn-shaped metamaterial structures (Figure 3c,d), which is due to the difficulty in achieving precise structural control during the manufacturing process. In this work, a FcFNN is used to estimate the units’ critical proportion required for the metamaterial to achieve its intended function. Although the metamaterials prepared by the bottom-up method have significant structural defects, the results show that they can work effectively as long as the critical proportion is met. Therefore, despite the numerical differences, the negative refraction characteristics are essentially consistent, and the computational results are instructive for loss optimization. This guidance is particularly applicable to the preparation of quasi-periodic metamaterials through chemical synthesis methods, significantly improving the tolerance and reliability of the preparation process. In cases where precise manufacturing of 3D structures cannot be achieved, a potential solution to reduce such discrepancies is to optimize the structure through inverse design, rather than merely focusing on the proportion of critical units, and this issue is worth further investigation in future work.

**Figure 5:**
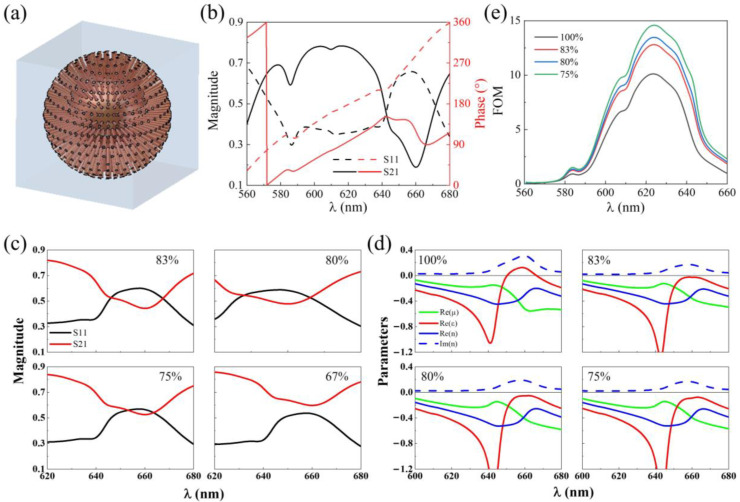
Optimization design of red light meta-cluster. (**a**) Schematic diagram of red light meta-cluster model. (**b**) simulated transmission and reflection spectra. (**c**) the transmission and reflectance spectra of the red light meta-cluster model with 83%, 80%, 75%, and 67% silver layer distributions. (**d**) effective electromagnetic parameters for the green light meta-cluster model with 100%, 83%, 80%, and 75% silver layer distributions. (**e**) FOM corresponding to (**d**).

Figure 5a,b depict the red light meta-cluster model, along with the simulated transmission and reflection spectra. Similar to the green light model, we retrieved the transmission and reflection spectra at silver distribution ratios of 83%, 80%, 75%, and 67%, as shown in Figure 5c. When the ratio is lower than 75%, the transmission and reflection curves separate and the correct effective electromagnetic parameters cannot be extracted (Figure 5d). The largest FOM was obtained for a distribution ratio of 75% (Figure 5e). The critical ratio of the red light meta-cluster model (75%) is slightly lower than that of the green light meta-cluster model (80%).

Similar to the previous model calculations, the ball-thorn-shaped structure still satisfies the disordered state design and the critical range selection. The critical range is near 80% for the green light model and 75% for the red light model, based on which the properties of the meta-cluster metamaterials can be optimally designed. Although the critical ratio of the meta-cluster metamaterials is higher than that of the split-ring model and the PAMAM-Ag model, the meta-cluster metamaterial model itself is optimally designed, and its distribution of discrete surface silver in the whole unit accounts for a much lower ratio than that of the other models. Based on the optimized design of the ball-thorn-shaped model, we have made the first measurements of negative refraction and inverse Doppler effect at visible light frequencies using the developed meta-cluster samples [28].

Based on the weak interaction theory of metamaterials [41,42,43], we can design a model of ball-thorn-shaped metamaterials that operates over a broad visible light spectrum as well as simulate the effective parameters. Similar to the single-frequency ball-thorn-shaped metamaterial, the effective electromagnetic parameters and the maximum FOM of the models with different silver distribution ratios can be obtained, which will greatly facilitate the design and preparation of broadband metamaterials. The broadband ball-thorn-shaped metamaterials were obtained by mixing eight meta-clusters with transmission peaks at 490 nm, 500 nm, 540 nm, 570 nm, 600 nm, 640 nm, 680 nm, and 700 nm (Appendix C) [29]. We have experimentally realized the negative refraction and inverse Doppler effect across most regions of the visible light spectrum for the first time. These anomalous optical properties are difficult to observe experimentally in the visible light wideband. In addition, using the broadband metamaterial, we experimentally obtained the trapped rainbow effect that slows down the speed of light and the black hole phenomenon that stops light [30]. One of the reasons why slowing down light has been difficult to realize for a long time is the high loss in slow light devices, i.e., insufficient caching time or insufficient length of light–matter interactions due to losses [47]. Different from the previously reported single rainbow, this amber rainbow band (i.e., a broadband light-trapping system) significantly enhances the interaction of light with matter, and the perfect back reflection that occurs after the incident angle reaches a critical value leads to an optical black hole that perfectly absorbs the energy outflow radiation. The amber rainbow effect exhibits not only the automatic frequency-selective response predicted by single-frequency theoretical models, but also the spatial periodic modulation induced by broadband omnidirectional visible metamaterials prepared by disordered assembly systems [30]. This disordered metamaterial can be expediently prepared in large areas by the bottom-up method, which opens the door for the preparation of 3D ultra-low loss metamaterials in the broadband of the visible spectrum. The broadband inverse Doppler effect is expected to be used to design a new type of radar with multi-frequency radiation sources, and the metamaterial layer based on the inverse Doppler effect can cause radar detection interference and cannot be locked. Slow optical waveguides based on these metamaterials allow broadband and room temperature operation, subverting traditional slow wave methods based on resonance or periodic configurations above the diffraction limit, and promising applications with light storage and energy harvesting. The critical proportion for metamaterials to execute the expected capabilities can be rapidly obtained through forward-predictive deep learning networks, which greatly saves costs and enhances efficiency. The unit structure design thus acquired provides a theoretical basis for optimizing experimental preparation schemes and determining the composition range of metamaterials. Recently, significant progress has been made in large-scale inverse design of photonic devices [48,49]. The introduction of large-scale inverse design technology is expected to further advance the optimization process of metamaterial performance and achieve innovative research on photonic devices.

## 5. Conclusions

In summary, we delved into the analysis of many types of metamaterials to devise an innovative strategy for optimizing metamaterial loss. Evolving from the typical “wire and split-ring” model to the 3D ball-thorn-shaped model, we have conducted computational analyses based on the deep learning concept and the principle of weak interactions in metamaterials. The computational results provide theoretical validation for the anomalous behavior of disordered discrete metamaterial systems and confirm the critical conditions necessary for the emergence of anomalous behaviors in various structural unit models. We have discovered that disordered discrete metamaterial systems are capable of generating anomalous behaviors, provided that their distribution ratios align with the critical range criteria. The outcomes of our research have yielded optimized design paradigms for both single-frequency and broadband metamaterials, particularly focusing on loss-related anomalous behavior. The validation of these findings paves the way for refined material design strategies, offering not only experimental flexibility but also theoretical scaffolding. This is especially pertinent to the fabrication of quasi-periodic metamaterials through chemical synthesis methods, where our findings can significantly enhance the tolerance and reliability of the preparation process.

## Data Availability

The data presented in this study are available on request to corresponding author.

## Appendix A

Numerical simulations: The metamaterial unit cell is solved using the time domain solver in CST Microwave Studio 2021 (Darmstadt, Germany). The boundary conditions of the model are set to perfect electric conductor (PEC) in the *x*-direction and perfect magnetic conductor (PMC) in the *y*-direction, and the light source is incident normally from the *z*-direction [28]. The effective electromagnetic parameters are extracted from the transmission and reflection spectra of the simulation results.

## Appendix B

Principle of disordered metamaterials: Unlike the collective resonance of PhC with a strong periodic structure, metamaterials are weakly interacting structures and the behavior of the system is revealed independently by each unit [41,43]. The periodicity and arrangement of the system units have little effect on its behavior. The automatic frequency-selective properties exhibited by the weak interactions provide a physical basis for the disordered assembly of metamaterials [28,29].

## Appendix C

Principle of broadband samples: Based on the weak interaction foundation of locally resonant elements [41,43], the individual units of a system resonate at the intrinsic frequency, and the total response of the system is a combination of different types of single resonance spectra. In the preparation of broadband samples, the units at different wavelengths are automatically self-assembled and randomly distributed in a disordered manner [29], and we only need to determine the appropriate content of these units without considering their geometrical occupancy.
